# Rac1 Impairs Forgetting-Induced Cellular Plasticity in Mushroom Body Output Neurons

**DOI:** 10.3389/fncel.2020.00258

**Published:** 2020-08-25

**Authors:** Isaac Cervantes-Sandoval, Ronald L. Davis, Jacob A. Berry

**Affiliations:** ^1^Department of Biology, Georgetown University, Washington, DC, United States; ^2^Interdisciplinary Program in Neuroscience, Georgetown University, Washington, DC, United States; ^3^Department of Neuroscience, The Scripps Research Institute Florida, Jupiter, FL, United States

**Keywords:** *Drosophila*, forgetting, olfactory memory, output neurons, memory trace, Rac1

## Abstract

Active memory forgetting is a well-regulated biological process thought to be adaptive and to allow proper cognitive functions. Recent efforts have elucidated several molecular players involved in the regulation of olfactory forgetting in *Drosophila*, including the small G protein Rac1, the dopamine receptor DAMB, and the scaffold protein Scribble. Similarly, we recently reported that dopaminergic neurons mediate both learning- and forgetting-induced plasticity in the mushroom body output neuron MBON-γ2α′1. Two open questions remain: how does forgetting affect plasticity in other, highly plastic, mushroom body compartments and how do genes that regulate forgetting affect this cellular plasticity? Here, we show that forgetting reverses short-term synaptic depression induced by aversive conditioning in the highly plastic mushroom body output neuron MBON-γ1pedc>α/β. In addition, our results indicate that genetic tampering with normal forgetting by inhibition of small G protein Rac1 impairs restoration of depressed odor responses to learned odor by intrinsic forgetting through time passing and forgetting induced acutely by shock stimulation after conditioning or reversal learning. These data further indicate that some forms of forgetting truly erase physiological changes generated by memory encoding.

## Introduction

Animals live in an exceptionally dynamic and noisy environment. Every day, organisms experience a plethora of sensory inputs but just a small fraction of these will remain as memories, and forgetting is the fate of the vast majority. We can infer that forgetting is advantageous in at least four different ways. First, as mentioned, we are exposed to an overwhelming amount of information. If memories were high-fidelity records of this information, we might soon saturate the capacities of the brain. Nevertheless, there seems to be copious space to store new information. This space may be provided continuously by active forgetting. Second, a system that consistently eliminates obsolete information would be more efficient in recalling useful memories. The lack of forgetting may make our memories as noisy as the environment itself. Third, forgetting may engender behavioral flexibility. Inflexible memory maintenance is incompatible with behavioral flexibility; a network strong at maintaining memories will be inferior at acquiring updating information. Finally, forgetting may be required for generalization. Memories are not precise representations of the past, but more akin to models of the past. Memories may have a better predictive value without the particulars of a determined experience, allowing for generalizations to be made (Richards and Frankland, 2017).

The *Drosophila* mushroom bodies (MB) are the main brain structure that is believed to be the coding center of olfactory memories (Heisenberg et al., 1985; Dubnau et al., 2001; McGuire et al., 2001); consequently, MB are also the site where molecular regulation of forgetting occurs (Shuai et al., 2010, 2011, 2015; Berry et al., 2012, 2015; Cervantes-Sandoval et al., 2016; Himmelreich et al., 2017; Zhang et al., 2018). During olfactory memory acquisition, positive or negative values to initially neutral odors are assigned by reinforcement. This reinforcement is achieved by the coincident activation of a sparse number of Kenyon cells (KC) by odorant and dopaminergic neurons (DAN) that innervate discrete zones, composed of 15 tile-like compartments of the MB lobes. Each of these tiles has a corresponding mushroom body output neuron (MBON), the activation of which favors either approach or avoidance behavior (Aso et al., 2014b; Owald et al., 2015). The molecular detection of the coincidence (Zars et al., 2000; McGuire et al., 2003; Tomchik and Davis, 2009) is thought to change the output weight of KC synapses onto the corresponding MBON (KC > MBON), suggesting a model in which dopamine-induced plasticity tilts the overall MBON network to direct appropriate behavior (Owald et al., 2015; Owald and Waddell, 2015). In fact, recent studies have shown that learning alters odor drive to specific MBONs (Séjourné et al., 2011; Pai et al., 2013; Plaçais et al., 2013; Bouzaiane et al., 2015; Hige et al., 2015; Owald et al., 2015; Perisse et al., 2016).

We have started to understand the molecular and circuit bases for memory forgetting regulation in *Drosophila*. We envision a model where not only does dopamine convey the reinforcement signal during memory acquisition (Schwaerzel et al., 2003; Schroll et al., 2006; Claridge-Chang et al., 2009; Aso et al., 2010, 2012; Liu et al., 2012; Burke et al., 2012; Handler et al., 2019), mediated by the dDA1 receptor, but continued-ongoing activity of the same DA neurons also promotes forgetting (Berry et al., 2012, 2015, 2018). This ongoing activity is regulated by the behavioral state of the animal, being highly active during locomotion and conversely quiescent during resting states (Berry et al., 2015). Continuous dopamine release from this activity presumably stimulates the DAMB receptor, which is only modestly required for learning but necessary for normal memory loss (Berry et al., 2012; Cervantes-Sandoval et al., 2016). The DAMB receptor is preferentially coupled to Gαq, and its activation mobilizes an intracellular signaling pathway scaffolded by Scribble that includes small G protein Rac1 and cofilin, all of which have been involved in the regulation of normal memory forgetting (Shuai et al., 2010; Cervantes-Sandoval et al., 2016; Himmelreich et al., 2017). Likewise, five different autism-associated proteins (Fmr1, Ube3a, Nlg4, Nrx1, and Tsc1) that fail to activate Rac1 also show defects in retroactive interference-induced forgetting (Dong et al., 2016). More recently, it was reported that in addition to the *DAMB* > *Gaq* > *Scribble* > *Rac1* > *Cofilin* pathway, there is an additional independent forgetting molecular pathway including raf/MAPK/NMMII. The authors suggested that these two forgetting pathways are additive and that they account for the regulation of the entire labile memory decay (Zhang et al., 2018).

Despite our growing understanding of the molecular and circuit regulation of memory forgetting, some fundamental questions remain: how does forgetting affect plasticity of highly plastic mushroom body compartments and how do genes that regulate forgetting affect this cellular plasticity? As mentioned above, learning induces strong plasticity changes that alter the odor drive to specific MBONs, and we recently found that this plasticity is bidirectionally regulated by dopamine neurons (Berry et al., 2012); here, we investigate the progression of a well-characterized memory trace formed in MBON-γ1pedc>α/β (Hige et al., 2015; Perisse et al., 2016) after aversive olfactory conditioning. We performed calcium transients imaging in live animals before and after aversive olfactory conditioning and after memory decay and acute induced forgetting due to interfering-electric shock or reversal learning. Finally, we compare the physiological changes in this MBON in flies with genetic insults that impaired memory forgetting. Our results indicate that, as previously shown, a mild aversive training induces a strong memory trace in MBON-γ1pedc>α/β, manifested as a depression of olfactory responses to the trained odor (CS+). The response to CS+ is restored to preconditioning responses, 30 min after training, by interfering electric shocks or by reversal training. Furthermore, overexpression of the dominant negative form of Rac1 (*Rac1^*N*17^*) dampens normal plasticity recovery.

## Materials and Methods

### *Drosophila* Husbandry

Flies were cultured on standard medium at room temperature. Crosses, unless otherwise stated, were kept at 25°C and 70% relative humidity with a 12 h light–dark cycle. The drivers used in this study include *MB112C-splitgal4* (Aso et al., 2014a), *R12G04-lexA* (Jenett et al., 2012), and *R13F02-gal4* (Jenett et al., 2012). Additional transgene stocks included *uas-GCaMP6f* (Chen et al., 2013), *lexAop-GCaMP6f* (Chen et al., 2013), *uas-rac^*N*17^* (Luo et al., 1994), and *tub-gal80^*ts*^* (McGuire et al., 2003). When the target system was used to restrict expression of transgene to adult animals, fly crosses were kept at 18°C during development. After eclosion of 1–2-day old flies, the were kept at 30°C for 4 days for the induction of expression. Flies were then kept at 25°C for 1 h before imaging. Control flies were subjected to exactly the same protocol, but they did not contain the UAS transgene. Additional controls were performed by keeping the crosses at 18°C for the entire time; these flies were then kept at 25°C for 1 h before imaging. We notice that the expression of lexAop-*GCaMP6f* was very low at 18°C, which makes imaging particularly difficult.

### *In vivo* Calcium Imaging

For measuring calcium responses with conditioning, odor, or shock delivery, we processed flies as previously described with some modifications (Cervantes-Sandoval et al., 2017). Briefly, a single fly was aspirated without anesthesia into a narrow slot the width of a fly in a custom-designed recording chamber. The head was immobilized by gluing the eyes to the chamber using myristic acid and the proboscis fixed to reduce movements. A small square section of the dorsal cuticle was removed from the head to allow optical access to the brain. Fresh saline [103 mM NaCl, 3 mM KCl, 5 mM HEPES, 1.5 mM CaCl_2_, 4 mM MgCl_2_, 26 mM NaHCO_3_, 1 mM NaH_2_PO_4_, 10 mM trehalose, 7 mM sucrose, and 10 mM glucose (pH 7.2)] was perfused immediately across the brain to prevent desiccation and ensure the health of the fly. Using a 25 × water-immersion objective and a Leica TCS SP5 II confocal microscope with a 488 nm argon laser, we imaged the MBON-γ1pedc>α/β neuron for 2 min, during which stimuli was delivered starting at 30 s after imaging initiation. We used one PMT channel (510–550 nm) to detect *GCaMP6s* fluorescence.

### Odor and Shock Presentation

To deliver odors to flies under the microscope, a small stream of air (500 ml/min) was diverted (via solenoids) from flowing through a clean 20 ml glass vial to instead flow through a 20 ml glass vial containing a 0.5 μl drop of pure odorant. This air stream was then serial diluted into a larger air stream (1,500 ml/min) before traveling through Teflon tubing (∼2.5 mm diameter) to reach the fly. To deliver shocks to flies under the microscope, a custom shock platform was made from shock grids used in standard olfactory memory assays that consist of alternating ± charged copper strips attached to an epoxy sheet. To simulate shock exposure given during the standard olfactory memory assay, the surface of the shock platform was positioned so that all six legs are touching but the fly could temporarily break contact by moving its legs. Both solenoids for odors control and Grass stimulator for shock presentation were controlled by Arduino microcontroller (Arduino Uno) with custom-made programs.

### Training Under Microscope Programs

The regular training protocol followed in most experiments on the paper, flies were presented to preconditioning odors with 5 s odor 1 followed 30 s of air and by 5 s of a second odor (non-associated odor) pretraining (MCH and OCT). Five min after this preconditioning recordings, flies were trained under the microscope by simultaneous presentation of a single 20 s odor pulse and four 90 V, 1.25 s electric shocks (5 s inter shock interval). Five, ten, or fifteen min after training, post-conditioning odor responses were recorded similarly to preresponses. This allowed recording odor responses right after conditioning as well as odor responses as memory decay progresses. For control purposes, flies were trained with either mock training, which consisted in the same protocol as before excluding the electric shock, or backwards training in which electric pulses were presented right before the onset of odor delivery.

In addition to normal memory decay, forgetting was acutely induced by two different protocols: shock interference (Berry et al., 2015; Aso and Rubin, 2016) and reversal learning (Shuai et al., 2010). For the former, flies were trained as previously but 12 1.25 s, 90 V shocks were presented 2 min after training and before post-conditioning odor recordings. For reversal learning flies were trained by presenting 20 s of CS+ odor along with electric shocks followed by 30 s of air and then 20 s of a second odor as CS−; 1 min after training, flies were retrained with the reverse odor contingency (CS− was now presented as CS+ and vice versa). Post-conditioned responses were recorded as previously.

### Quantification and Statistical Analysis

Fluorescence was acquired from a region of interest (ROI) drawn around the axon tract of MBON-γ1pedc>α/β. Baseline was calculated using a Matlab code as the mean fluorescence across the 5 s before each odor presentation. This baseline was then used to calculate %ΔF/Fo for the complete recording. Boxplots represent distribution of %ΔF/Fo responses across the 5 s of odor presentation. Solid lines in fluorescence traces represent mean %ΔF/Fo ± SE (shaded area) across the odor responses.

Statistics were performed using Prism 5 (GraphPad). All tests were two tailed and confidence levels were set at α = 0.05. The figure legends present the *p*-values and comparisons made for each experiment. Unless otherwise stated, non-parametric tests were used for all imaging data.

## Results and Discussion

New insights have demonstrated that associative olfactory learning changes the output weight of KC synapses onto the corresponding MBON, suggesting a model in which dopamine-induced plasticity tilts the overall MBON network to direct appropriate behavior (Owald et al., 2015; Owald and Waddell, 2015). In fact, recent physiological studies have shown that learning alters odor drive to specific MBONs (Séjourné et al., 2011; Pai et al., 2013; Plaçais et al., 2013; Bouzaiane et al., 2015; Hige et al., 2015; Owald et al., 2015; Perisse et al., 2016; Felsenberg et al., 2017; Berry et al., 2018). As a whole, these changes can be described as memory traces (Davis, 2011). Interestingly, reward learning appears to reduce the drive to output pathways that direct avoidance, whereas aversive learning increases drive to avoidance pathways while reducing the drive to approach pathways. We choose to explore how forgetting and its genetic disruption affected memory traces formed in MBON-γ1pedc>α/β. We selected this trace because it can be easily induced after a very short stimulation of odor (1 s) along with optogenetic stimulation of dopaminergic neuron innervating the same MB compartment (Hige et al., 2015). In addition, Aso and Rubin (2016) showed, using optogenetics, and behavior, that MB-γ1 compartment is the fastest to encode new memories, the most unstable or susceptible to memory decay and shock interference. Furthermore, they showed that memories in this compartment are highly vulnerable to retroactive interference induced by a formation of additional olfactory memory. These features increased our chances to observe forgetting related changes after memory encoding.

Using electrophysiology whole-cell recordings of MBON-γ1pedc>α/β, Hige et al. (2015) showed that pairing and odor with specific artificial activation of dopaminergic PPL1-γ1pedc induced odor-specific synaptic depression. In addition, in 2016, Perisse et al. (2016) showed that training the flies by pairing 1 min of CS+ with 12 shocks followed by 1 min presentation of CS−, induced a decreased response to the CS+ relative to CS− compared to no change in mock trained animals. Here, we first try to confirm that this depression is observed when individual flies are imaged before and after learning and is observable using calcium reporter *GCaMP6f*. For this, we trained the fly under a confocal microscope as indicated in “Materials and Methods” and recorded calcium responses to odors in MBON-γ1pedc>α/β before and after training using split-gal4 driver *MB112c*. Supplementary Figure S1B showed that pairing 20 sec of MCH presentation with electric shock delivered to the fly legs by a floating electric grid platform induced a robust depression of calcium response to the learned odor. This depression was specific to the paired odor and was not observed in OCT, which was used as a non-paired odor. Additionally, this decreased response was not observed when flies were trained by a mock training (no shock) (Supplementary Figure S1C) or backwards training (shock presented before odor onset) (Supplementary Figure S1D). Finally, training flies with the reciprocal odor (OCT) showed similar results (Supplementary Figure S1E). These results confirm previous results and demonstrated that aversive olfactory conditioning induces under the microscope induced a robust memory trace represented as a depression of MBON-γ1pedc>α/β calcium responses to trained odors.

**FIGURE 1:**
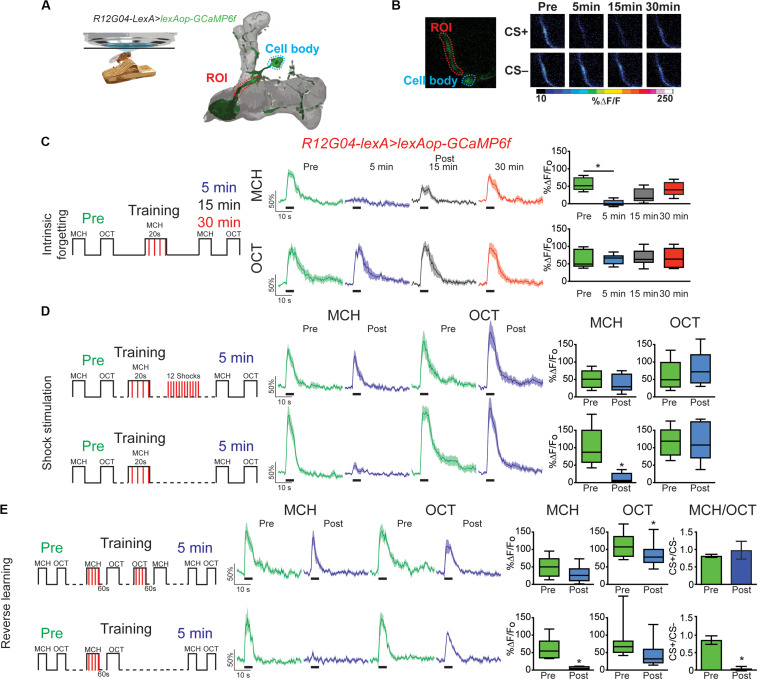
Forgetting reverses memory-induced plasticity in MBON-γ1pedc>α/β. **(A)** Diagram of *in vivo* under the microscope training and imaging setup and MBON-γ1pedc>α/β. **(B)** Pseudocolored peak responses of MBON-γ1pedc>α/β to CS + (MCH) and CS− (OCT) before and after paired training **(C)** Left, diagram of experimental setup: preconditioning responses were obtained for MCH and OCT; later flies were aversively trained to MCH and 5, 15, and 30 min later post-conditioning responses were recorded. MCH responses were completely depressed 5 min after training. These responses recovered from depression and were no longer significant from preresponses 15 and 30 min after training. Non-parametric Friedman test *p* = 0.0013; Dunn’s multiple comparison, *p < 0.001, *n* = *8.* **(D)** Left, diagram of experimental setup: flies were trained as before except 12 electric shocks were inserted after training and before post-odor responses (upper plots). Simultaneously, as experimental control, flies were also trained as before but excluding the interfering-electric shocks (lower plots). No significant changes were detected to either MCH or OCT when electric shocks were presented, Wilcoxon paired test, *p* > 0.0547, n = 8. In contrast MCH was completely depressed in control conditions, Wilcoxon paired test, *p* = 0.0039, *n* = 8. **(E)** Left, diagram of experimental setup: flies were trained as indicated; reversal training was inserted after training and before post-odor responses (upper plots). Simultaneously, as experimental, control, flies were also trained as before but excluding the interfering-reversal learning (lower plots). No significant changes were detected to either MCH or OCT when reversal learning was introduced. In contrast MCH was completely depressed in control conditions Wilcoxon paired test, *p > 0.0156, *n* = 7. Difference between Pre and Post CS+/CS− ratios was not significant after reversal learning, non-parametric Wilcoxon paired test, *p* = 0.9375, *n* = 7. In contrast, difference between Pre and Post CS+/CS− ratios was significant in control conditions, non-parametric Wilcoxon paired test, *p = 0.0156, *n* = 7. Boxplots represent distribution of %ΔF/Fo responses across the 5 s of odor presentation. The thick black bar below each trace represent the time of odor presentation.

Next, we ask how is this memory trace affected when forgetting occurs either intrinsically (as time passes) or is induced by interfering-electric shock or reversal learning. For this experiment we expressed *GCaMP6f* in MBON- γ1pedc>α/β using *R12G04-lexA* driver. Flies were trained as above and post-training responses were recorded 5, 15, or 30 min after conditioning. Similar to prior result, full depression to learned odor was observed 5 min after conditioning. This depression showed increasing recovery and was no longer significant from preconditioning responses 15 or 30 min after training (Figures 1B,C). No significant changes were detected in the non-paired odor (OCT). This data demonstrate that at least for the memory trace observed in MBON-γ1pedc>α/β under these training conditions, intrinsic forgetting restitutes MCH calcium responses to normal levels after 30 min. It is important to indicate that Perisse et al. (2016) showed that the decreased response to CS+ observed after 1 min CS+ odor pairing, lasted for at least 3 to 4 h after training. These differences, of course, could be attributed to the fact that we used a reduced training protocol (20s pairing) intending to improve our chances of detecting rapid changes in the observed plasticity.

We have previously shown that mechanical stimulation mediated by dopaminergic neurons can promote forgetting if presented after learning (Berry et al., 2015). Similarly, Aso and Rubin (2016) showed a decrease in conditioned response as a result of DAN activation after artificially induced aversive learning. These results suggested a model where dopamine bidirectionally regulates connectivity between KC > MBON; this regulation would be contingent on dopamine release in the context of odor presentation or not. Here we ask whether the presentation of electric shock pulses presented after learning restored memory trace observed in MBON-γ1pedc>α/β to preconditioning levels. Results indicate that 12, 90 V shocks presented after conditioning was enough to restore responses to the paired MCH odor back to preconditioning levels (Figure 1D). Responses to the non-paired odor were not affected by any of the protocols followed (Figure 1). Additionally, presenting the four shocks alone after conditioning was not enough to restore responses to CS+ (Supplementary Figure S2), indicating that the effect of shocks alone and reversal learning are somehow different.

Inducing acute memory forgetting can also be achieved by retroactive interference. In flies, it has been demonstrated that training with reversal conditioning, where flies are trained by presenting a first odor paired with electric shock followed by a second non-paired odor as a CS− and then immediately trained with the reverse contingency, showed decrease memory performance to the first CS+ (Shuai et al., 2010; Cervantes-Sandoval et al., 2016; Dong et al., 2016). Here we showed that retroactive interference induced forgetting by reversal learning also restored MCH responses to preconditioning levels (Figure 1E). Responses to OCT after reversal conditioning were scarcely significantly decreased. Additionally, analysis of the CS+/CS− ratio showed that reversal conditioning not only restored responses to initial associated odor but also interfered with the synaptic depression of the newly learned contingency, namely no difference between responses ratios pre and post-conditioning (Figure 1E). These results contrast with findings in plasticity induced in MBON-γ2 α′1, where reversal learning restores responses to initial CS+ and simultaneously depresses responses to the new CS+ (Berry et al., 2018). Here our results suggest the presence of not only retroactive interference to initial memory but also forgetting of secondary memory induced by proactive interference, which has been previously reported behaviorally (Cervantes-Sandoval et al., 2016). This difference can be attributed to the fact that different MB domains have different properties (Aso and Rubin, 2016).

**FIGURE 2:**
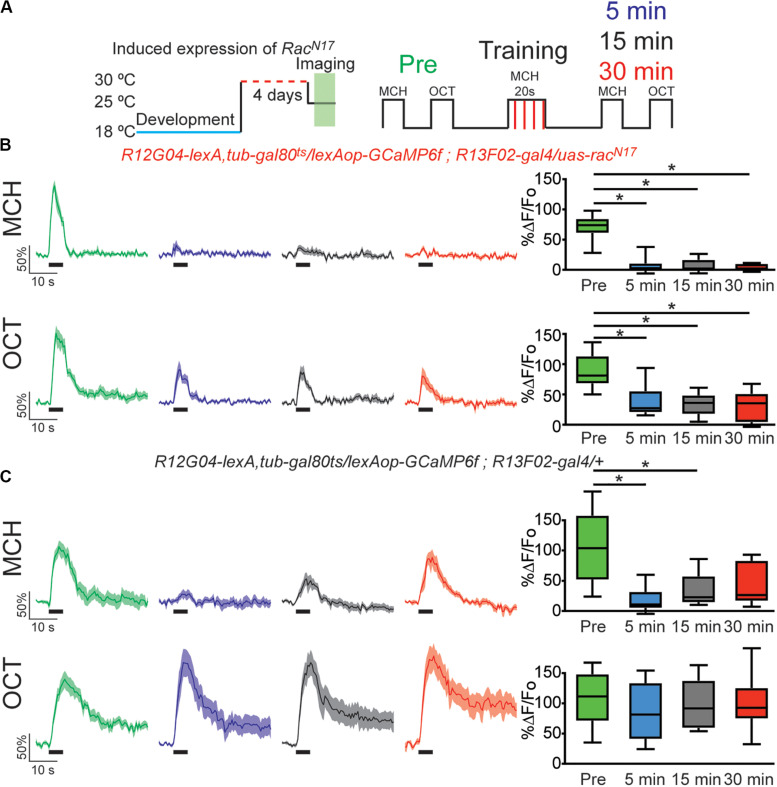
Expression of dominant negative form of Rac1 (*Rac^*N*17^*) in KC hindered restoration of depressed odor responses. **(A)** Diagram of temperature and training schedule: Expression of *Rac^*N*17^* in KC was induced by shifting 1–2 day old flies from 18 to 30°C for 4 days. Flies were then shifted to 25°C 1 h before functional imaging. Control flies contained all genetic elements but *uas*-*rac^*N*17^* transgene. Preconditioning responses were obtained for MCH and OCT; later flies were aversively trained to MCH and 5, 15, and 30 min later post-conditioning responses were recorded. **(B)** MCH responses, in experimental flies, were completely depressed 5 min after training. These responses remained completely depressed 15 and 30 after training. Non-parametric Friedman test *p* < 0.001; Dunn’s multiple comparison, *p < 0.0021. *n* = 9. A partial but significant depression was also observed in non-paired odor (OCT). Non-parametric Friedman test *p* < 0.076; Dunn’s multiple comparison, *p < 0.032. *n* = 9. Two-way Anova and Sidek multiple comparisons test between CS+ and CS− responses of experimental group showed that depression observed in CS + is higher to the non-specific depression observed in the CS− responses (Pre responses, *p* = 0.5222; 5 min responses, *p = 0.0223; 10 min responses, *p = 0.0073; 15 min responses, *p = 0.0496). **(C)** MCH responses, in control flies, were also completely depressed 5 min after training. These responses showed recovery and were no longer different from preresponses 30 min after training. Non-parametric Friedman test *p* < 0.0003; Dunn’s multiple comparison, *p < 0.032. *n* = 9. No significant depression was observed in non-paired odor (OCT). Boxplots represent distribution of %ΔF/Fo responses across the 5 s of odor presentation. The thick black bar below each trace represent the time of odor presentation.

In 2010 Shuai et al. described one of the central molecular regulators of active forgetting, the small G protein Rac1; they found that overexpression of dominant negative (DN) form of Rac1 (*Rac^*N*17^*) impairs normal memory forgetting. We decided to test how the memory trace in MBON-γ1pedc>α/β is affected by genetic disruption of this active forgetting regulation. For this we trained flies that express *GCaMP6f* on MBON-γ1pedc>α/β using lexA driver, *R12G04-lexA*, while expressing DN form of Rac1 in KC using gal4 driver *R13F02-gal4*. Expression of *Rac^*N*17^* in KC was further confined to adulthood using target system (see “Materials and Methods”). Flies expressing *RacN*^17^ expression in adulthood showed a normal complete depression to the learned MCH odor. Nevertheless, these flies showed impaired recovery of memory-induced plasticity in MBON-γ1pedc>α/β after 15 and 30 min after conditioning with MCH (Figure 2B). Unexpectedly, we observed a mild non-specific depression to the non-paired odor. This non-specific depression might be a result of Rac1 inhibition broadening odors representation and therefore increase in generalization; other explanations might also be possible. Despite this, two-way Anova analysis with Sidak’s multiple comparisons test showed that the depression observed to the paired odor is significantly higher that the non-specific depression to the CS−.

Control flies carrying all genetic insertion but the *uas-Rac^*N*17^* and subjected to the same temperature conditions, showed normal depression to the learned odor and full recovery of odor response 30 min after conditioning (Figure 2C). Control flies did not show a depressed odor response to the non-paired odor. Flies kept at 18°C to keep target system at non-permissive temperature showed normal learning-induced odor depression as well as normal recovery of odor calcium responses (Supplementary Figure S3). These results suggest that, at least partially, *Rac^*N*17^* inhibits forgetting by impairing the bidirectional regulation of KC > MBON plasticity that is to say the restoration of the depressed odor responses to CS+ (MCH) in MBON-γ1pedc>α/β.

**FIGURE 3:**
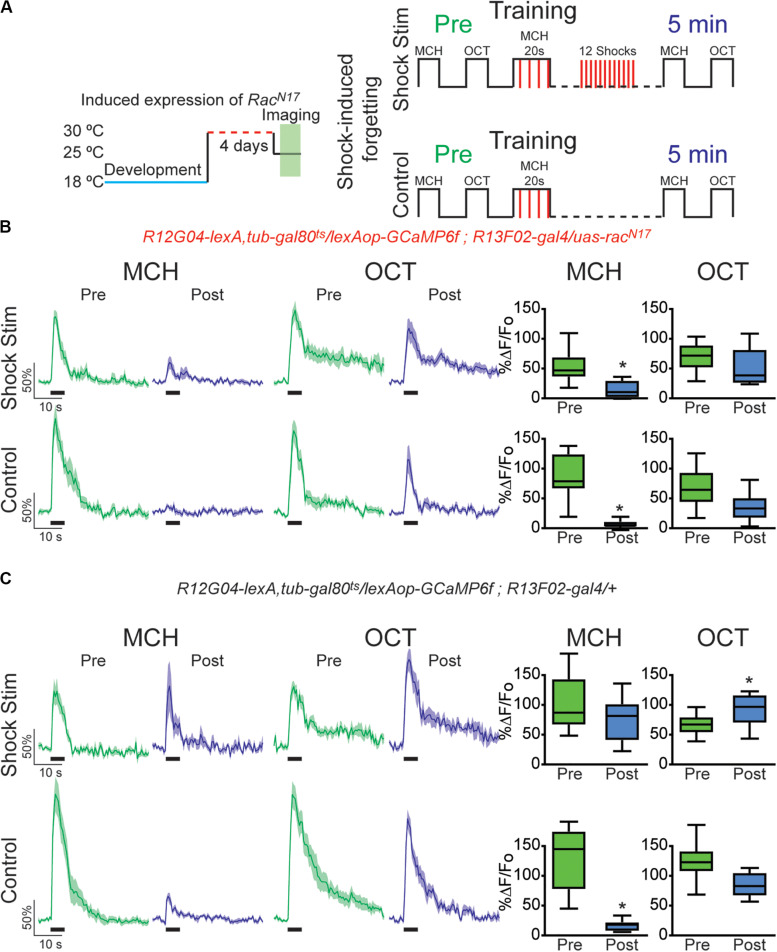
Expression of dominant negative form of Rac1 (*Rac^*N*17^*) in KC hindered restoration of depressed odor responses after shock-induced forgetting. **(A)** Diagram of temperature and training schedule: Expression of *Rac^*N*17^* in KC was induced by shifting 1–2-day-old flies from 18 to 30°C for 4 days. Flies were then shifted to 25°C 1 h before functional imaging. Control flies contained all genetic elements but *uas*-*rac^*N*17^* transgene. Preconditioning responses were obtained for MCH and OCT; later flies were aversively trained to MCH and 5 min later post-conditioning responses were recorded. For experimental condition, flies received 12, 90 V interfering-shocks (shock stim) between training and testing. For control condition, flies receive no interfering electric shocks. **(B)** MCH responses, in both control and shock stim conditions, were depressed 5 min after training in flies expressing *Rac^*N*17^* in KC, non-parametric Wilcoxon paired test, *p = 0.0039, *n* = 9. No significant depression was observed in non-paired odor (OCT) after shock stim conditions, non-parametric Wilcoxon paired test, *p* = 0.3008, *n* = 9. As previously observed a mild significant depression to OCT was observed in control conditions, non-parametric Wilcoxon paired test, *p = 0.0391, *n* = 9. **(C)** MCH responses in flies containing no *uas-rac^*N*17^* transgene were completely restored to preconditioning levels after shock stimulation as recorded 5 min after training, non-parametric Wilcoxon paired test, *p* = 0.1953, *n* = 8. As expected, in the control condition, responses to MCH were completely depressed, non-parametric Wilcoxon paired test, *p = 0.0078, *n* = 8. A significant increase was observed in non-paired odor (OCT) in shock stim conditions, non-parametric Wilcoxon paired test, *p = 0.032, *n* = 8. Additionally, a small decrease in responses to non-paired odor was observed in control conditions, non-parametric Wilcoxon paired test, *p = 0.0078, *n* = 8. Boxplots represent distribution of %ΔF/Fo responses across the 5 s of odor presentation. The thick black bar below each trace represents the time of odor presentation.

The above results indicate that the recovery of depressed olfactory responses in MBON-γ1pedc>α/β to a learned odor mediated by intrinsic forgetting, or normal memory decay trough time passing, is impaired when the DN form of *Rac1* is expressed in KC. Next, we sought to investigate if *Rac^*N*17^* also affected memory trace loss when this is induced by interfering-electric shocks presented after learning. For that we trained flies as before and then we deliver 12, 90 V electric shocks to fly legs to induce acute forgetting. Flies expressing DN form of *Rac^*N*17^* in KC showed no recovery in learned-odor calcium responses (MCH) as compared to control flies (Figures 3B,C). These results indicated that genetically interfering with memory forgetting by the expression of DN *Rac^*N*17^* in KC impairs not only intrinsic forgetting but also acute dopamine-mediated forgetting induced by strong electric shock stimulation after learning.

**FIGURE 4:**
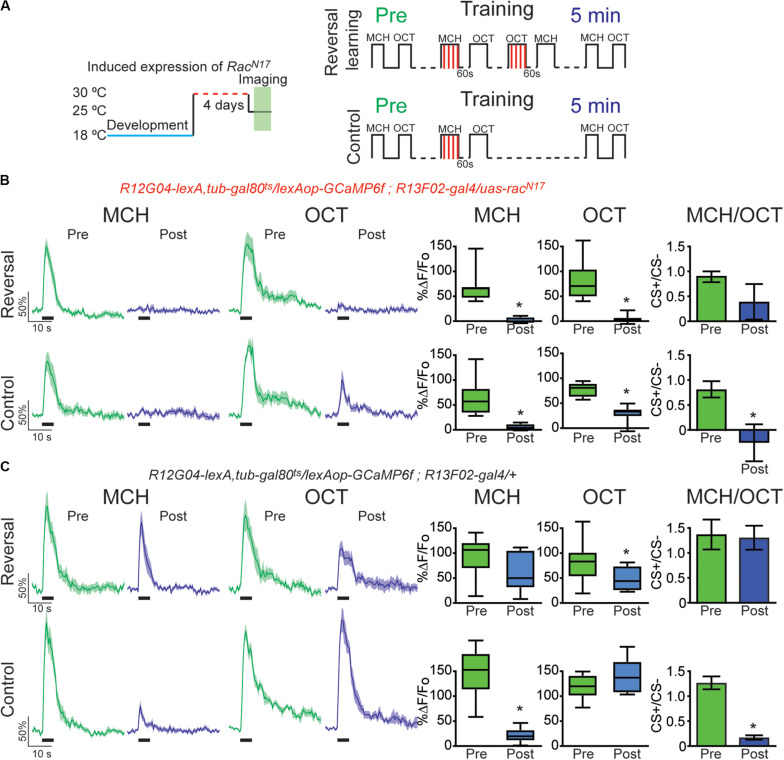
Expression of the dominant negative form of Rac1 (*Rac^*N*17^*) in KC hindered restoration of depressed odor responses after reversal learning-induced forgetting. **(A)** Diagram of temperature and training schedule: Expression of *Rac^*N*17^* in KC was induced by shifting 1 day old flies from 18 to 30°C for 4 days. Flies were then shifted to 25°C for 1 h before functional imaging. Control flies contained all genetic elements but *uas*-*rac^*N*17^* transgene. Preconditioning responses were obtained for MCH and OCT; later flies were aversively trained to MCH, and 5 min later, post-conditioning responses were recorded. For the experimental condition, flies received a reversal learning (reversal) between training and testing. For the control condition, flies received no reversal learning. **(B)** MCH responses, in both control and reversal learning conditions, were depressed 5 min after training in flies expressing *Rac^*N*17^* in KC, non-parametric Wilcoxon paired test, *p ≤ 0.0156, *n* = 8. A complete depression was also observed to OCT after reversal conditioning, non-parametric Wilcoxon paired test, *p = 0.0078. A partial but significant depression was also observed in non-paired odor (OCT) in control conditions, non-parametric Wilcoxon paired test, *p* = 0.0156, *n* = 8. The difference between Pre and Post CS+/CS− ratios was not significant after reversal learning, non-parametric Wilcoxon paired test, *p* = 0.1953, *n* = 8. In contrast, the difference between Pre and Post CS+/CS− ratios was significant in control conditions, non-parametric Wilcoxon paired test, *p = 0.0312, *n* = 8. **(C)** MCH responses in flies containing no *uas-rac^*N*17^* transgene were completely restored to preconditioning levels after reversal learning, non-parametric Wilcoxon paired test, *p* = 0.1484, *n* = 8. As expected, in the control condition, responses to MCH were completely depressed, non-parametric Wilcoxon paired test, *p = 0.0078, *n* = 8. A mild significant depression was observed to OCT after reversal conditioning, non-parametric Wilcoxon paired test, *p = 0.0391. No significant depression was observed in non-paired odor (OCT), non-parametric Wilcoxon paired test, *p* = 0.3828, *n* = 8. The difference between Pre and Post CS+/CS− ratios was not significant after reversal learning, non-parametric Wilcoxon paired test, *p* = 0.9553, *n* = 8. In contrast, the difference between Pre and Post CS+/CS− ratios was significant in control conditions, non-parametric Wilcoxon paired test, *p = 0.0078, *n* = 8. Boxplots represent distribution of %ΔF/Fo responses across the 5 s of odor presentation. The thick black bar below each trace represents the time of odor presentation.

Finally, we investigated the effects of DN *Rac^*N*17^*expression on retroactive interference forgetting provoked by reversal conditioning. For this, we trained the flies presenting a first odor paired with electric shock (MCH, CS+) followed by air and a second odor not paired with electric shock (OCT, CS−). After learning flies were subjected to reversal training in which previous CS− odor was now paired with electric shock and the former conditioned odor was now presented as CS−. This protocol acutely induced a complete recovery of cellular memory trace in MBON- γ1pedc>α/β in control animals (Figure 4C). Surprisingly, once again analysis of CS+/CS− ratio showed that reversal conditioning not only restored responses to initial associated odor but also interfered with the synaptic depression of the newly learned contingency, namely no difference between responses ratios pre and post conditioning (Figure 4C). Again, these results reinforce the suggestion of proactive interference (Cervantes-Sandoval et al., 2016). Expression of *Rac^*N*17^* in KC during adulthood not only impaired memory trace restoration of initial contingency but also induced a strong depression to the secondary paired odor (Figure 4B). These results indicated that genetically interfering with memory forgetting by expression of DN *Rac^*N*17^* in KC impairs restoration of olfactory responses in MBON-γ1pedc>α/β induced by intrinsic memory loss (time passing) (Figure 2), acute forgetting induced by a non-associative stimuli (electric shock) (Figure 3), and acute forgetting by new associations or memory updating (reversal learning) (Figure 4).

## Concluding Remarks

Our study indicates that forgetting reverses synaptic depression induced by aversive conditioning in MBON-γ1pedc>α/β. This is true for intrinsic memory forgetting through time passing, and acute forgetting by both interfering-electric shock and retroactive interference provoked by reversal learning. Our results also show physiological evidence of proactive interference in MBON-γ1pedc>α/β, previously observed behaviorally in *Drosophila* (Cervantes-Sandoval et al., 2016), where prior learning interferes with the formation of new learning. Results also indicate that genetic tampering with normal forgetting by inhibition of small G protein Rac1 impairs restoration of depressed odor responses to learned odor by the three mechanisms described above. It has been recently reported that Rac1 partially regulates forgetting through time passing as well as forgetting induced by reversal learning but it does not affect forgetting induced by non-associative experiences like heat stress, electric shocks or odor presented alone (Zhang et al., 2018). Our results indicate that at least at physiological level Rac1 inhibition does affect odor responses restoration induced by electric shock in MBON-γ1pedc>α/β. It is possible that this apparent discrepancy is due to the fact that here we only explore the memory trace of a single MBON whereas, as mentioned before, behavior rises, most likely, as a combinational effect of the whole KC > MBON network. Therefore, a single compartment analysis does not necessarily reflect final behavior. It is also important to indicate that in this study, we used a reduced training protocol (20 s odor with four shocks) when compared to the classical training paradigm used for behavioral studies (1 min odor with 12 shocks). We used this mild training session to increase our chances of observing the reversal of synaptic plasticity. It remains to be studied, the dynamic of these physiological changes when flies are trained with classical 1-min protocol. It was recently reported that training flies with a single training cycle (1 min odor presentation along with 12 shocks) induces an independent contextual memory that resides in the lateral horn. It is very likely that the forgetting described in this study and others have different dynamics and/or rules to this context-dependent memory (Zhao et al., 2019).

In memory research, one school of thought holds that nothing is ever lost from storage and that forgetting represents only a temporal failure or inhibition of access to memory. The other school holds that memory is not completely preserved and that forgetting is a true erasure of information from storage (Davis and Zhong, 2017). Our findings, previous (Berry et al., 2012) and here, indicate that normal forgetting reverses plasticity generated by aversive learning in MBON-γ1pedc>α/β suggesting that forgetting, in the case of short-term non-protein-synthesis dependent memories, truly erases at least some of physiological changes caused by memory encoding. This finding does not exclude the possibility that other compartments have different properties nor that the same phenomenon is true for long-term memories. It is possible that memories that had undergone protein-synthesis dependent memory consolidation are more resistant to permanently reverse the physiological changes that form part of the long-term memory trace. In that case, when talking about forgetting, we could not talk about erasure but rather a transient blockage of memory retrieval.

## Data Availability Statement

The datasets generated for this study are available on request to the corresponding author.

## Author Contributions

RD, JB, and IC-S contributed to the design of the study, interpreted the results, and contributed to the final version of the manuscript. JB and IC-S implemented the experimental paradigms. IC-S performed the experiments, analyzed the data, and wrote an initial draft of the manuscript. All authors contributed to the article and approved the submitted version.

## Conflict of Interest

The authors declare that the research was conducted in the absence of any commercial or financial relationships that could be construed as a potential conflict of interest.

## Supplementary Material

FIGURE S1 Flies trained under the microscope form a calcium-based memory trace in MBON-γ1pedc>α/β. **(A)** Diagram of in vivo under the microscope training and imaging setup. **(B)** Left, diagram of experimental setup: preconditioning responses were obtained for MCH and OCT in flies expressing *GCaMP6f* using the split-gal4 driver MB112C; later, flies were aversively trained to MCH and post-conditioning responses were recorded 5 min later. MCH responses are completely depressed 5 min after aversive conditioning, non-parametric Wilcoxon-paired test, **p* = 0.0156; in contrast non-paired odor presented no change, non-parametric Wilcoxon-paired test, *p* = 0.1562. **(C)** Left, diagram of experimental setup: flies were processed as in A but electric shock was excluded. No significant changes in odor responses to MCH or OCT were detected, non-parametric Wilcoxon-paired test, *p* ≤ 0.4375. **(D)** Left, diagram of experimental setup: Flies were trained as in A but electric shocks were presented before odor onset. No significant changes in odor responses to MCH or OCT were detected, non-parametric Wilcoxon-paired test, *p* = 0.2969. **(E)** Left, diagram of experimental setup: preconditioning responses were obtained for MCH and OCT; later flies were aversively trained to OCT and 5 min later post-conditioning responses were recorded. OCT responses are completely depressed 5 min after aversive conditioning, non-parametric Wilcoxon-paired test, **p* = 0.0312; in contrast non-paired odor presented no change, non-parametric Wilcoxon-paired test, *p* = 0.6875. *n* = 6–7 for all experiments. Boxplots represent distribution of %ΔF/Fo responses across the 5 s of odor presentation. The thick black bar below each trace represent the time of odor presentation.

FIGURE S2 Left, diagram of experimental setup: flies were trained as indicated, then 4 shock pulses were inserted after training and before post-odor responses (upper plots). As positive control, flies were trained as before but 12 shocks were presented after training and before post-odor responses (lower plots). No significant changes were detected to either MCH or OCT when 12 shocks were introduced. Wilcoxon-paired test, *p* ≥ 0.3125. In contrast MCH was completely depressed when only 4 shocks were presented. Wilcoxon-paired test, **p* > 0.0078, *n* = 8. No changes were detected in OCT responses. Wilcoxon-paired test, *p* = 0.3828, *n* = 8. Boxplots represent distribution of %ΔF/Fo responses across the 5 s of odor presentation. The thick black bar below each trace represent the time of odor presentation.

FIGURE S3 Forgetting reverses memory-induced plasticity in MBON-γ1pedc>α/β. **(A)** Diagram of temperature and training schedule: Expression of *Rac*^*N*17^ in KC was delimited by keeping 1–2 day old flies at 18°C for 4 days. Flies were then shifted to 25°C 1 h before functional imaging. Control flies contained all genetic elements but *uas-rac*^*N*17^ transgene. Pre-conditioning responses were obtained for MCH and OCT; later flies were aversively trained to MCH and 5, 15 and 30 min later post-conditioning responses were recorded. **(B)** MCH responses, in experimental genotype, were completely depressed 5 min after training. These responses were restored to preconditioning levels 30 after training. No significant depression was observed in non-paired odor (OCT). Non-parametric Friedman test, *p* = 0.0002. Dunn’s multiple comparison, **p* < 0.032, *n* = 7. **(C)** MCH responses, in control genotype, were completely depressed 5 min after training. These responses showed recovery and were no longer different from pre-responses 30 after training. No significant depression was observed in non-paired odor (OCT). Non-parametric Friedman test, *p* = 0.0001. Dunn’s multiple comparison, **p* < 0.032, *n* = 8. The thick black bar below each trace represent the time of odor presentation.
