# Supplementary figures and images for: Rac1 Impairs Forgetting-Induced Cellular Plasticity in Mushroom Body Output Neurons
Source: Front Cell Neurosci. 2020 Aug 25;14:258. doi: 10.3389/fncel.2020.00258 (PMC7477079; doi:10.3389/fncel.2020.00258)

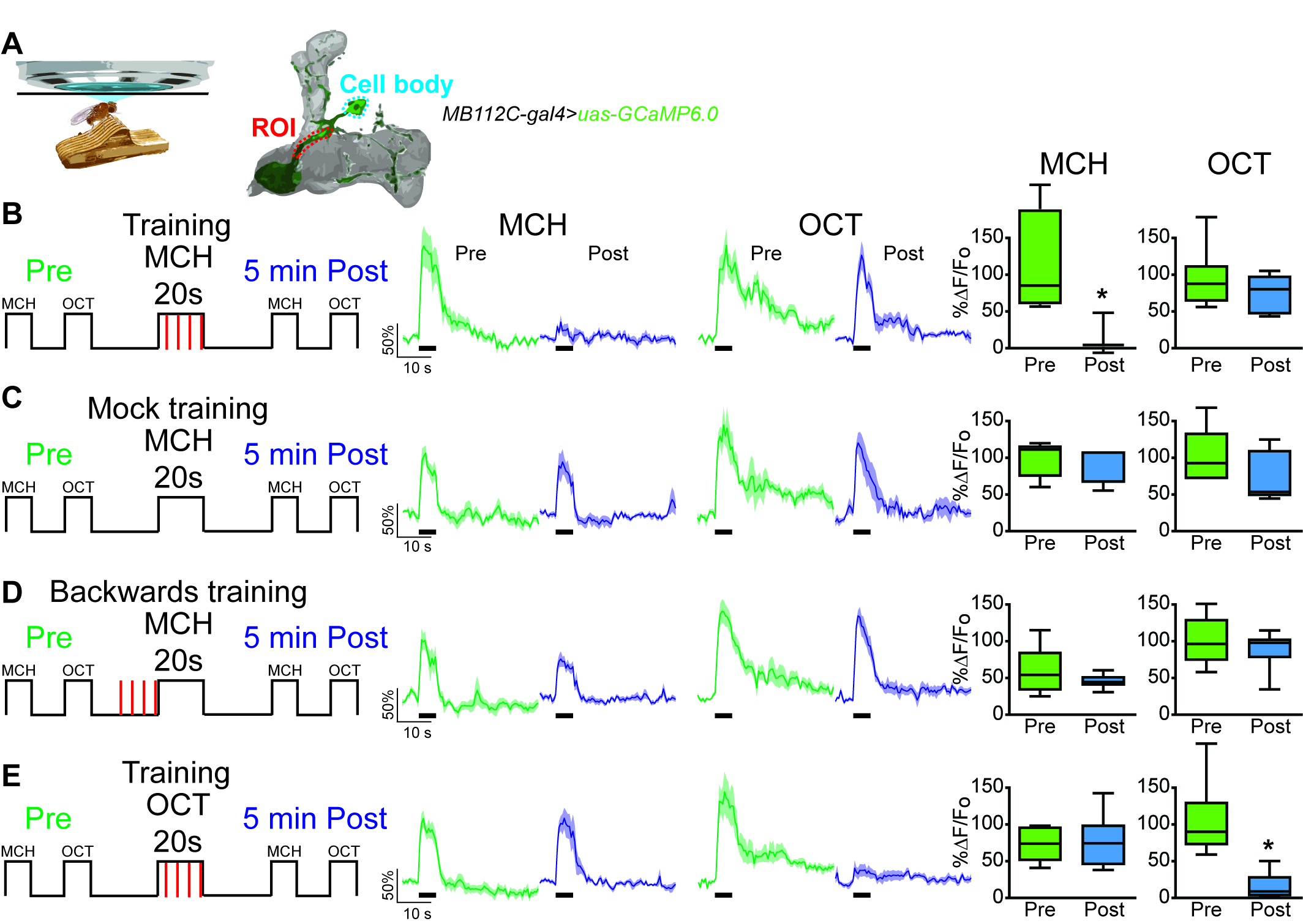

Supplement: FIGURE S1 — Flies trained under the microscope form a calcium-based memory trace in MBON-γ1pedc>α/β. (A) Diagram of in vivo under the microscope training and imaging setup. (B) Left, diagram of experimental setup: preconditioning responses were obtained for MCH and OCT in flies expressing GCaMP6f using the split-gal4 driver MB112C; later, flies were aversively trained to MCH and post-conditioning responses were recorded 5 min later. MCH responses are completely depressed 5 min after aversive conditioning, non-parametric Wilcoxon-paired test, *p = 0.0156; in contrast non-paired odor presented no change, non-parametric Wilcoxon-paired test, p = 0.1562. (C) Left, diagram of experimental setup: flies were processed as in A but electric shock was excluded. No significant changes in odor responses to MCH or OCT were detected, non-parametric Wilcoxon-paired test, p ≤ 0.4375. (D) Left, diagram of experimental setup: Flies were trained as in A but electric shocks were presented before odor onset. No significant changes in odor responses to MCH or OCT were detected, non-parametric Wilcoxon-paired test, p = 0.2969. (E) Left, diagram of experimental setup: preconditioning responses were obtained for MCH and OCT; later flies were aversively trained to OCT and 5 min later post-conditioning responses were recorded. OCT responses are completely depressed 5 min after aversive conditioning, non-parametric Wilcoxon-paired test, *p = 0.0312; in contrast non-paired odor presented no change, non-parametric Wilcoxon-paired test, p = 0.6875. n = 6–7 for all experiments. Boxplots represent distribution of %ΔF/Fo responses across the 5 s of odor presentation. The thick black bar below each trace represent the time of odor presentation. [file Image_1.tif]

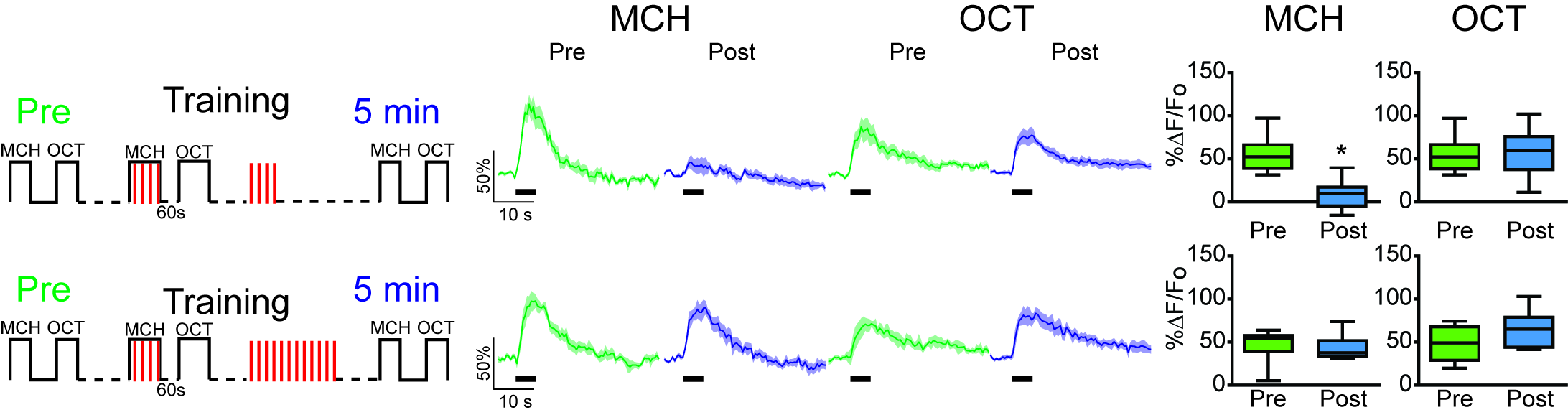

Supplement: FIGURE S2 — Left, diagram of experimental setup: flies were trained as indicated, then 4 shock pulses were inserted after training and before post-odor responses (upper plots). As positive control, flies were trained as before but 12 shocks were presented after training and before post-odor responses (lower plots). No significant changes were detected to either MCH or OCT when 12 shocks were introduced. Wilcoxon-paired test, p ≥ 0.3125. In contrast MCH was completely depressed when only 4 shocks were presented. Wilcoxon-paired test, *p > 0.0078, n = 8. No changes were detected in OCT responses. Wilcoxon-paired test, p = 0.3828, n = 8. Boxplots represent distribution of %ΔF/Fo responses across the 5 s of odor presentation. The thick black bar below each trace represent the time of odor presentation. [file Image_2.tif]

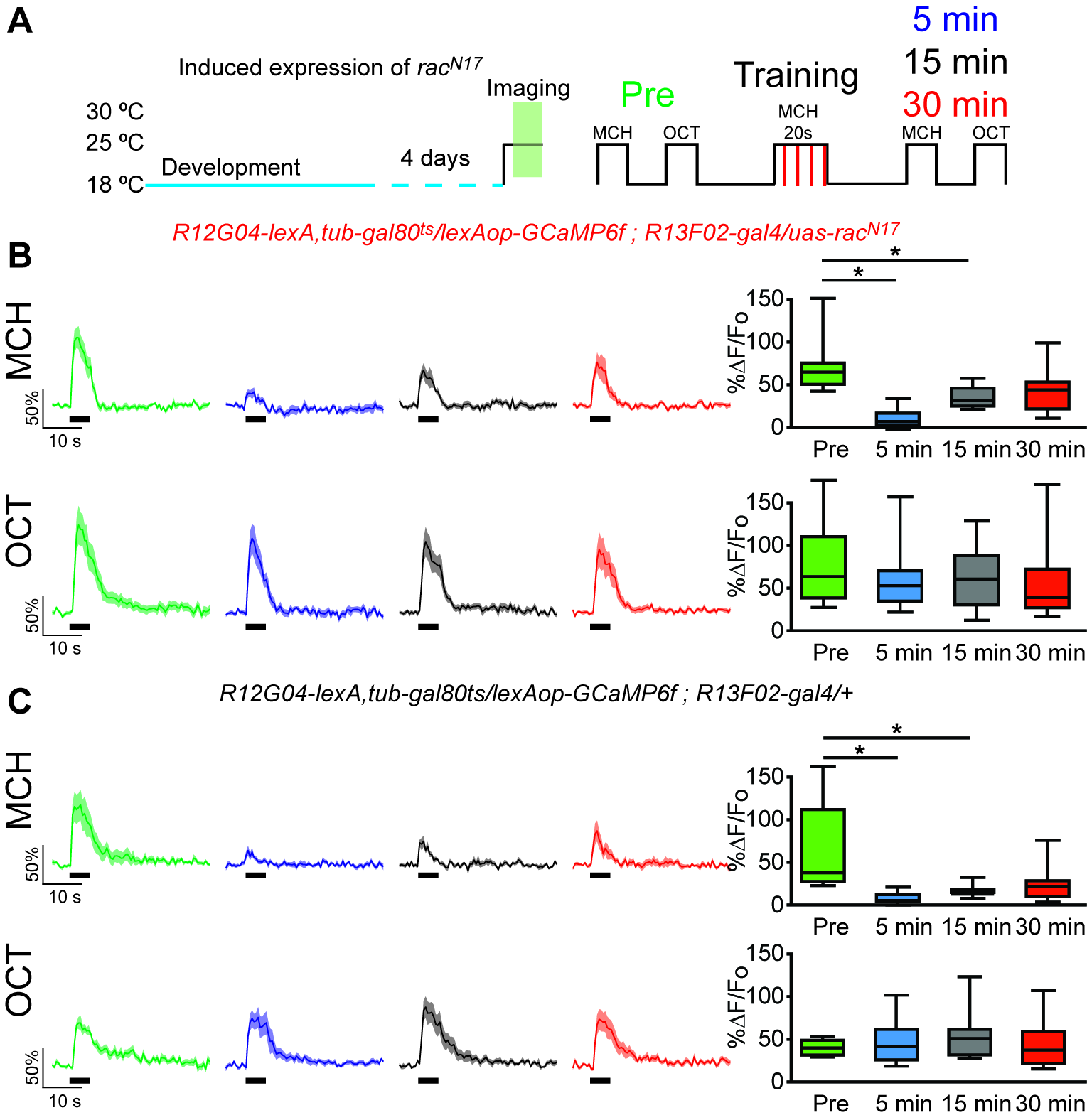

Supplement: FIGURE S3 — Forgetting reverses memory-induced plasticity in MBON-γ1pedc>α/β. (A) Diagram of temperature and training schedule: Expression of RacN17 in KC was delimited by keeping 1–2 day old flies at 18°C for 4 days. Flies were then shifted to 25°C 1 h before functional imaging. Control flies contained all genetic elements but uas-racN17 transgene. Pre-conditioning responses were obtained for MCH and OCT; later flies were aversively trained to MCH and 5, 15 and 30 min later post-conditioning responses were recorded. (B) MCH responses, in experimental genotype, were completely depressed 5 min after training. These responses were restored to preconditioning levels 30 after training. No significant depression was observed in non-paired odor (OCT). Non-parametric Friedman test, p = 0.0002. Dunn’s multiple comparison, *p < 0.032, n = 7. (C) MCH responses, in control genotype, were completely depressed 5 min after training. These responses showed recovery and were no longer different from pre-responses 30 after training. No significant depression was observed in non-paired odor (OCT). Non-parametric Friedman test, p = 0.0001. Dunn’s multiple comparison, *p < 0.032, n = 8. The thick black bar below each trace represent the time of odor presentation. [file Image_3.tif]
