# Supplementary material for: Rac1 Impairs Forgetting-Induced Cellular Plasticity in Mushroom Body Output Neurons
Source: Front Cell Neurosci. 2020 Aug 25;14:258. doi: 10.3389/fncel.2020.00258 (PMC7477079; doi:10.3389/fncel.2020.00258)
Supplement: Supplementary file 4 [file Data_Sheet_1.PDF]

Table S1. Statistical details. Related to figures 1-4 and S1-S3.

Figure	Experiment	n	Statistical test	p-value
Figure 1C	Intrinsic forgetting CS+	n=8	Friedman test	$p=0.0013$
			Dunn's multiple comparison	Pre vs 5min, $p<0.001$<br>Pre vs 15min, $p>0.05$<br>Pre vs 30min, $p>0.05$
	Intrinsic forgetting CS–	n=8	Friedman test	$p=0.9564$
Figure 1D	Shock stimulation (experimental)	n=8	Wilcoxon-paired test	CS+, $p=0.0547$
				CS–, $p=0.0781$
	Shock stimulation (control)	n=8	Wilcoxon-paired test	CS+, $p=0.0039$
				CS–, $p=1$
Figure 1E	Reversal learning (experimental)	n=8	Wilcoxon-paired test	CS+, $p=0.0547$
				CS–, $p=0.0156$
	Reversal learning (control)	n=7	Wilcoxon-paired test	CS+, $p=0.0156$
				CS–, $p=0.0781$
Figure 2B	Intrinsic forgetting CS+	n=9	Friedman test	$p=0.001$
			Dunn's multiple comparison	Pre vs 5min, $p<0.0021$<br>Pre vs 15min, $p<0.0021$<br>Pre vs 30min, $p<0.0021$
	Intrinsic forgetting CS–	n=9	Friedman test	$p=0.0076$
			Dunn's multiple comparison	Pre vs 5min, $p<0.032$<br>Pre vs 15min, $p<0.032$<br>Pre vs 30min, $p<0.032$
Figure 2C	Intrinsic forgetting CS+	n=9	Friedman test	$p=0.0003$
			Dunn's multiple comparison	Pre vs 5min, $p<0.001$<br>Pre vs 15min, $p<0.032$<br>Pre vs 30min, $p>0.1234$
	Intrinsic forgetting CS–	n=9	Friedman test	$p=0.3561$
Figure 3B	Shock stimulation (Shock Stim)	n=9	Wilcoxon-paired test	CS+, $p=0.0039$
				CS–, $p=0.3008$
	Shock stimulation (control)	n=9	Wilcoxon-paired test	CS+, $p=0.0039$
				CS–, $p=1$
Figure 3C	Shock stimulation (experimental)	n=8	Wilcoxon-paired test	CS+, $p=0.1953$
				CS–, $p=0.0391$
	Shock stimulation (control)	n=8	Wilcoxon-paired test	CS+, $p=0.0078$
				CS–, $p=0.0078$
Figure 4B	Reversal learning	n=8	Wilcoxon-paired test	CS+, $p=0.0078$
				CS–, $p=0.0078$
	No reversal learning	n=8	Wilcoxon-paired test	CS+, $p=0.0156$
				CS–, $p=0.0156$
Figure 4C	Reversal learning	n=8	Wilcoxon-paired test	CS+, $p=0.1484$
				CS–, $p=0.0391$
	No reversal learning	n=8	Wilcoxon-paired test	CS+, $p=0.0078$
				CS–, $p=0.3828$
Figure S1B	Training MCH	n=7	Wilcoxon-paired test	CS+, $p=0.0156$
				CS–, $p=0.1562$
Figure S1C	Mock training	n=6	Wilcoxon-paired test	CS+, $p=0.8125$
				CS–, $p=0.4375$
Figure S1D	Backwards training	n=7	Wilcoxon-paired test	CS+, $p=0.2969$
				CS–, $p=0.3750$
Figure S1E	Training OCT	n=6	Wilcoxon-paired test	CS+, $p=0.0312$
				CS–, $p=0.6875$
Figure S2	4 Shocks	n=8	Wilcoxon-paired test	CS+, $p=0.0078$
				CS–, $p=0.3828$
	12 Shocks	n=8	Wilcoxon-paired test	CS+, $p=0.8432$
				CS–, $p=0.3125$
Figure S3B	Intrinsic forgetting MCH (18 °C)	n=7	Friedman test	$p=0.0002$
			Dunn's multiple comparison	Pre vs 5min, $p<0.001$<br>Pre vs 15min, $p<0.032$<br>Pre vs 30min, $p>0.1234$
	Intrinsic forgetting OCT (18 °C)	n=7	Friedman test	$p=0.1604$
Figure S3C	Intrinsic forgetting MCH (18 °C)	n=8	Friedman test	$p=0.0001$
			Dunn's multiple comparison	Pre vs 5min, $p<0.0001$<br>Pre vs 15min, $p<0.032$<br>Pre vs 30min, $p>0.1234$
	Intrinsic forgetting OCT (18 °C)	n=8	Friedman test	$p=0.2898$
